# Covalent Inhibitors Targeting Mycobacterial Enzymes: Current Status, Challenges and Future Perspectives

**DOI:** 10.3390/ph19050707

**Published:** 2026-04-30

**Authors:** Mariana Luiza Silva, Matteo Mori, Stefania Villa, Andrea Tresoldi, Fiorella Meneghetti, Marcelle de Lima Ferreira Bispo

**Affiliations:** 1 Laboratory for Synthesis of Medicinal Molecules (LaSMMed), Department of Chemistry, State University of Londrina, Rodovia Celso Garcia Cid (PR-445), Londrina 86057-970, Brazil; mariana.luiza@uel.br (M.L.S.); mlfbispo@uel.br (M.d.L.F.B.); 2 Department of Pharmaceutical Sciences, University of Milan, Via Luigi Mangiagalli 25, 20133 Milano, Italy; matteo.mori@unimi.it (M.M.); stefania.villa@unimi.it (S.V.); andrea.tresoldi@unimi.it (A.T.)

**Keywords:** covalent inhibitors, tuberculosis (TB), mycobacterial enzymes, anti-TB drug discovery

## Abstract

This review offers a critical and comprehensive overview of the most promising covalent inhibitors against traditional and emerging enzymatic targets of *Mycobacterium tuberculosis* (*Mtb*). Nearly three decades after the World Health Organisation’s (WHO) declaration of tuberculosis (TB) as a global health emergency, *Mtb* continues to claim millions of lives, remaining among the leading causes of death worldwide. In recent years, several efforts have been devoted to shortening and improving treatment outcomes and to overcoming increasing drug resistance. The aim of our work is to provide a perspective on recent progress in the field of covalent inhibitors of mycobacterial enzymes, highlighting the current landscape and outlining future directions for safer and more effective strategies.

## 1. Introduction

**Figure 1:**
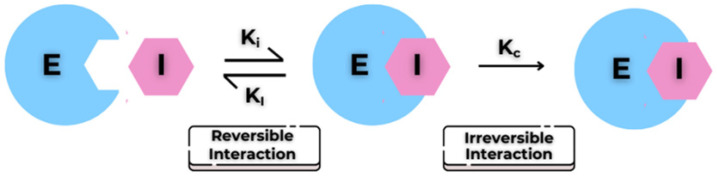
Reversible and irreversible interactions between the enzyme (E) and the inhibitor (I).

Covalent inhibitors are an important class of small molecules that inactivate their targets by forming a covalent bond, thereby generating a drug-target adduct that can be either irreversible or reversible. Unlike classical reversible inhibitors, which rely on transient non-covalent interactions, covalent inhibitors enable durable and sustained target suppression [1]. The mechanism of covalent enzymatic inhibition is typically described as a two-step process. Initially, the inhibitor binds non-covalently to the enzyme’s active site with an affinity expressed by the dissociation constant *K_I_* (or *K_i_*) (Figure 1). The electrophilic reactive group, commonly referred to as the “warhead”, is positioned near a suitably oriented nucleophilic residue, such as cysteine, serine, lysine, or tyrosine. Subsequently, a chemical reaction converts the reversible complex into a covalently modified enzyme; this is a time-dependent step, typically characterised by a *k_inact_*. The *k_inact_*/*K_I_* ratio provides a standard measure of covalent inhibitory efficiency, enabling potency comparisons across compound series [2,3].

The nature of the warhead-residue interaction determines whether inhibition is irreversible or reversible. Irreversible covalent bonds abolish enzymatic activity until de novo protein synthesis restores function, yielding a prolonged pharmacodynamic effect. Conversely, in reversible covalent inhibition, the covalent adduct can undergo bond cleavage (e.g., hydrolysis), allowing recovery of enzyme activity. Common electrophiles include epoxides, aziridines, acrylamides and other α,β-unsaturated carbonyls, haloacetamides, sulfonyl fluorides, nitriles, as well as reversible covalent moieties such as aldehydes/ketones and boronic acids [1]. Because the warhead governs reactivity, selectivity, and reversibility, its optimisation requires tuning intrinsic electrophilicity to maximise target binding while minimising non-specific modification of off-target proteins.

From a pharmacological point of view, covalent inhibitors can provide potent and long-lasting target suppression because, once a covalent adduct is formed, inhibition can persist even as free drug concentrations decline [4]. As a result, they can maintain robust target engagement under fluctuating exposure conditions, whereas reversible inhibitors may rapidly lose occupancy as drug concentrations decline [5]. Moreover, covalent inhibition can be advantageous for targets with shallow or poorly featured binding sites. By forming an irreversible (or slowly reversible) adduct with the target, covalent inhibitors enable effective modulation of proteins that are difficult to drug through non-covalent interactions [6].

Despite these advantages, covalent inhibitors entail liabilities related to electrophile reactivity. A primary concern is off-target covalent modification of proteins, and potentially other biomolecules, through reaction with endogenous nucleophiles, which can perturb cellular function and contribute to toxicity or adverse events. Another limitation is the requirement for a suitably positioned nucleophilic residue in the target binding site, which restricts the range of proteins amenable to covalent inhibition and introduces a potential route to resistance [5].

In the context of antimycobacterial drug discovery, the extended residence time achievable with covalent inhibitors may be particularly advantageous against slow-growing mycobacteria. This is especially relevant for *Mycobacterium tuberculosis* (*Mtb*), the causative agent of tuberculosis (TB), which remains a leading cause of mortality worldwide. Accordingly, there is strong motivation to explore covalent strategies targeting mycobacterial enzymes [7]. This review surveys recent advances in covalent compounds targeting mycobacterial enzymes and provides an overview of the current landscape while outlining future directions for the development of safer and more effective therapeutic strategies. Collectively, the findings presented here highlight the emerging potential of covalent inhibition as a viable approach in anti-TB drug discovery.

## 2. Enzymatic Targets and Covalent Inhibitors

### 2.1. Serine Hydrolases

**Figure 2:**
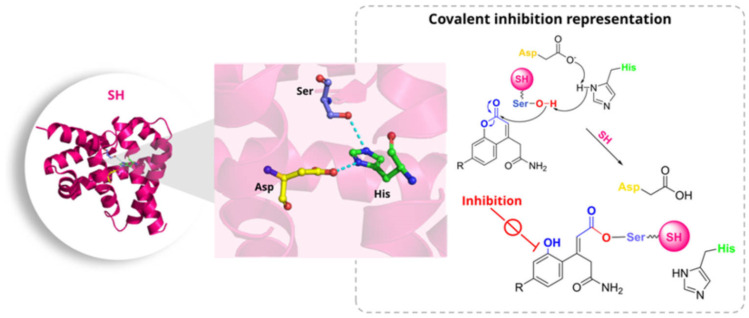
The left panel shows the three-dimensional structure of an SH, while the middle panel highlights the conserved Ser–His–Asp catalytic triad. The right panel depicts a representative mechanism of covalent inhibition, in which the inhibitor reacts with the hydroxyl group of the catalytic serine to form a stable covalent adduct, thereby blocking enzyme activity.

Mycobacterial serine hydrolases (SHs) constitute an extensive enzymatic superfamily, with approximately 70–80 predicted members in the *Mtb* genome. These enzymes play fundamental roles in cell envelope biogenesis and membrane remodelling, participating in the synthesis and degradation of triacylglycerols and complex lipids of the outer layer. These processes are essential for bacterial viability, persistence, and virulence. SHs are catalytically characterised by the Ser–His–Asp (or Ser–His–Glu) catalytic triad, in which the activation of the catalytic serine enables nucleophilic attack on the substrate, leading to the formation of a transient acyl-enzyme intermediate. This intermediate is subsequently hydrolysed, constituting an archetypal two-step process that defines the SH superfamily (Figure 2) [8,9]. Structurally, SHs adopt either an α/β-hydrolase or a protease-like fold, with the catalytic serine positioned within a conserved “nucleophilic elbow” and typically stabilised by an adjacent oxyanion hole. A significant proportion of these enzymes is localised in the periplasm or associated with the cell membrane. This structural positioning renders them particularly susceptible to direct inhibition by small-molecule compounds [10].

A functionally important subset of SHs is represented by serine proteases, enzymes dedicated to the hydrolysis of peptide bonds and increasingly implicated in mycobacterial virulence. Among this subfamily, TesA functions as a large thioesterase involved in the biosynthesis of outer-layer lipids, while LipG displays bifunctional phospholipase/thioesterase activity that contributes to envelope remodelling. Several additional lipases/esterases, including LipO, LipI, LipM, LipN, NlhH/LipH, and Cut2, have been identified as targets in activity-based screening using covalent inhibitors. Beyond the enzymes identified through activity-based profiling, the SH repertoire also includes a broader set of predicted esterases and lipases (Rv2079, Rv1400c, Rv2928, Rv2257c, Rv1192, Rv0646c, Rv1399c, Rv1426c and Rv2301), as well as functionally diverse serine-dependent complexes such as the caseinolytic protease ClpP1P2 and the Antigen 85 complex, an SH with additional transferase activity [10].

**Figure 3:**
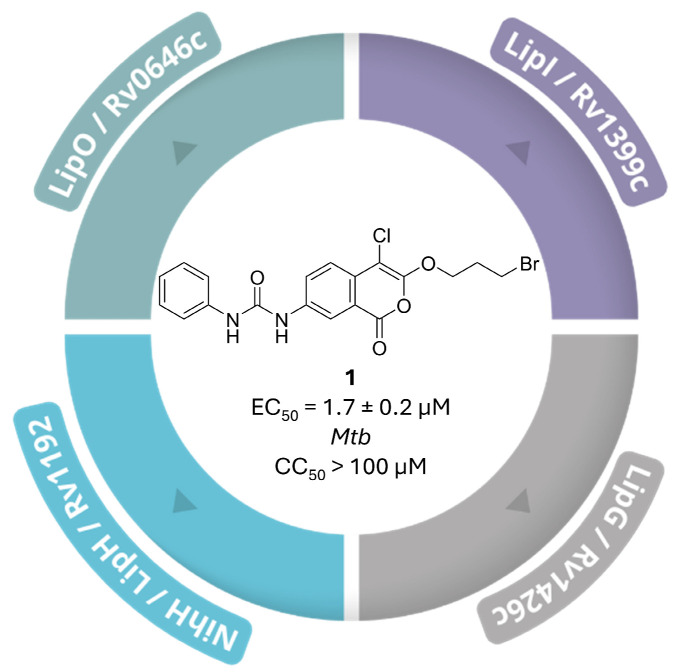
Activity of **1** against mycobacterial SHs, including LipO, LipI, LipG and NlhH/LipH enzymes.

In line with the growing interest in targeting these enzymes, phenotypic screening approaches have been employed to identify covalent inhibitors acting on this enzyme class. In 2022, Babin et al. conducted a screen of 942 electrophilic small molecules designed to react with catalytic serine residues, identifying 46 growth-inhibiting hits, predominantly belonging to two chemotypes: 7-urea chloroisocoumarins and diphenyl phosphonates. Seven compounds exhibited EC_50_ values < 5 µM without detectable host–cell toxicity; among them, **1** (Figure 3), a 7-urea chloroisocoumarin derivative, emerged as the most potent inhibitor of mycobacterial proliferation (EC_50_ = 1.7 ± 0.2 µM at 7 days; bacteriostatic profile; no cytotoxicity up to 100 µM in murine macrophages and human fibroblasts) [10].

Using competitive activity-based protein profiling (ABPP) with fluorescent and alkyne-tagged probes, the authors demonstrated that **1** covalently modifies nine SHs, including LipO, LipI, LipG and NlhH/LipH. To confirm that probe competition reflected true covalent adduct formation rather than transient target occupancy, they synthesised two alkyne-tagged analogues of the chloroisocoumarin warhead (**2**, EC_50_ = 8.7 ± 1.1 µM, and **3**, EC_50_ = 36 ± 11 µM; Table S1, Supplementary Materials) and applied live-cell click-labelling. Pre-incubation with **1** efficiently competed away four distinct bands, confirming a shared mechanism involving nucleophilic attack by active-site serines. LFQ-MS analysis further validated these nine SHs as bona fide covalent targets through significant signal reduction upon **1** pre-treatment. Finally, lipidomic profiling of treated cultures revealed accumulation of free mycocerosic, phthioceranic, and phthienoic acids, alongside a marked depletion of lipooligosaccharides, indicating that coordinated inhibition of these SHs disrupts the lipid metabolic pathways required for mycobacterial cell-wall biogenesis [10].

**Figure 4:**
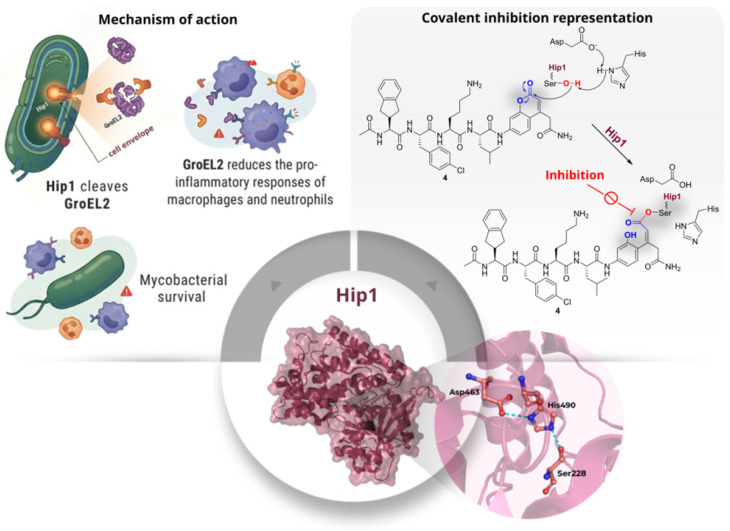
Hip1 mechanism of action. The left-upper panel summarises the proposed role of Hip1 in *Mtb* immune modulation: the enzyme cleaves GroEL2, and the processed GroEL2 reduces pro-inflammatory responses in macrophages and neutrophils, thereby promoting pathogen survival. The right-upper panel illustrates the mechanism of covalent inhibition, showing the stable covalent adduct, blocking enzyme activity.

Hip1 (Hydrolase Important for Pathogenesis/Rv2224c/MT2282) is a cell-envelope-associated serine protease of *Mtb* that plays a pivotal role in bacillary virulence. Hip1 has the capacity to modulate host inflammatory responses by cleaving the heat-shock protein GroEL2 at two distinct sites within its N-terminal region. The initial cleavage is preferentially driven by a lysine residue occupying the P2 position, which enhances substrate recognition and catalytic efficiency. This precise proteolytic processing of GroEL2 is essential for the ability of the pathogen to manipulate host immunity and sustain itself. Structurally, Hip1 adopts the conventional fold of SHs, featuring the Ser-His-Asp catalytic triad positioned in a nucleophilic elbow adjacent to the oxyanion hole, thereby enabling the formation of an acyl-enzyme intermediate during catalysis. Its localisation in the cell envelope ensures the facile accessibility of the enzyme to small-molecule inhibitors (Figure 4). Activity-based protein profiling studies have demonstrated that Hip1 can be selectively modified and inhibited by covalent probes, thereby confirming the druggability of its catalytic serine [10,11].

**Figure 5:**
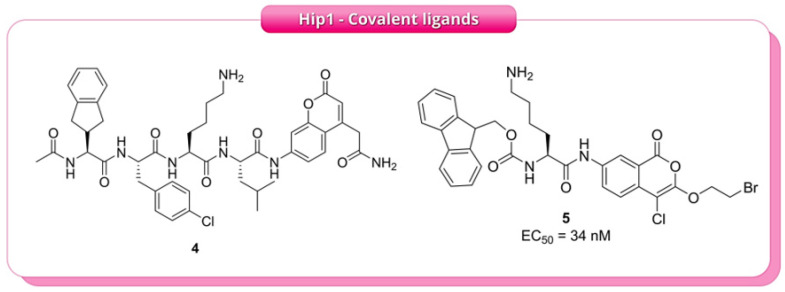
Chemical structures of the key covalent inhibitors reported by Lentz et al. (2016) [11]. Compound **4** represents the optimised fluorogenic substrate used to quantify Hip1 enzymatic activity, while compound **5** is a covalent chloroisocoumarin inhibitor demonstrating the most potent inhibition.

To define the substrate specificity of Hip1, Lentz et al. (2016) [11] utilised two complementary profiling approaches: positional scanning synthetic combinatorial libraries (PS-SCL) and multiplex substrate profiling by mass spectrometry (MSP-MS), both applied to the recombinant enzyme. The results, obtained using peptide substrates to probe protease specificity, indicated a consistent recognition pattern. Using the conventional P1–P4 nomenclature (defined relative to the scissile bond), Hip1 showed a strong preference for lysine at position P2, tolerance for aliphatic residues at P1, a preference for aromatic residues at P3, and limited selectivity at P4. These findings facilitated the design of an optimised fluorogenic substrate, **4** (Ac-Igl-4ClPhe-Lys-Leu-ACC, Figure 5), which exhibited an approximately 10,000-fold increase in catalytic efficiency compared to the reference substrate. Building on **4**, the authors screened a focused library of approximately 500 serine-reactive electrophiles, identifying a series of chloroisocoumarins as time-dependent inhibitors. Further optimisation produced compound **5** (Figure 5), featuring an Fmoc-l-Lys substituent at R1, which demonstrated an IC_50_ of 34 nM, exhibiting about 15-fold selectivity over human neutrophil elastase. Covalent modification of Hip1 by **5** was confirmed through SDS-PAGE, which showed a loss of FP-TMR labelling after pre-treatment with the inhibitor, indicating direct chemical modification rather than simple competitive displacement (Figure 5). Ultimately, substrate **4** enabled direct detection of Hip1 activity, while inhibitor **5** supported target validation through activity-based assays, demonstrating that substrate-profiling-guided design can produce selective chemical tools for monitoring and potentially modulating this virulence-associated enzyme [11].

**Figure 6:**
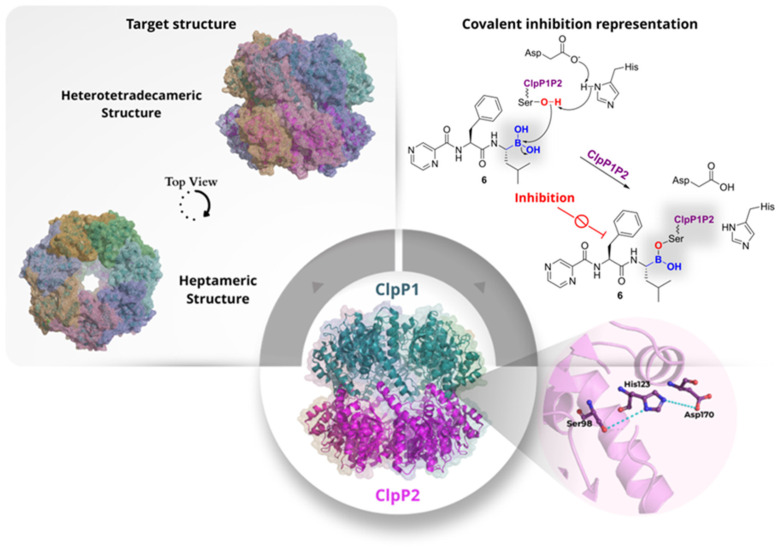
Structural organisation of the mycobacterial ClpP1P2 complex and mechanism of covalent inhibition. The left panel shows alternative oligomeric assemblies, depicted as surface representations in two views (heterotetradecameric and heptameric states). Each colour in the structure corresponds to a distinct subunit within the multimeric protease. The right panel shows how inhibitors react with the catalytic serine residues of ClpP1P2 to form stable covalent adducts that block proteolytic activity.

ClpP1P2 is a caseinolytic protease complex that functions to maintain protein homeostasis. The ATP-dependent ClpC1 or ClpX complex recognises substrates bearing specific degradation signals, including misfolded, stress-damaged, and regulatory proteins. These ATPases unfold the substrates and translocate them into the proteolytic chamber of ClpP1P2 for rapid degradation into short peptides. This process helps prevent toxic protein aggregation, modulate metabolic pathways, and aid adaptation to environmental stress. The complex is also involved in virulence mechanisms, being crucial for proteome remodelling during the non-replicating phase and important for the growth and survival of the pathogen. In *Mtb*, ClpP1P2 is a heterotetradecameric protein composed of a heptameric ClpP1 ring stacked on a heptameric ClpP2 ring (Figure 6). Each protomer contains an N-terminal gated pore, a head domain that forms the central barrel, and a “handle region” that mediates inter-ring contacts and correctly positions the catalytic triad (His-Ser-Asp). Beyond its structural organisation, the complex operates as a conformational switch that toggles between inactive and active forms according to the state of its handle region. In the compact, inactive conformation (T-state), the handle is disordered, and the catalytic triad is misaligned, so substrate degradation is inefficient. The binding of specific activating peptides or covalent inhibitors stabilises the handle, aligns the catalytic residues, and elongates the proteolytic chamber, thereby turning the enzyme on (R-state) [12,13].

**Figure 7:**
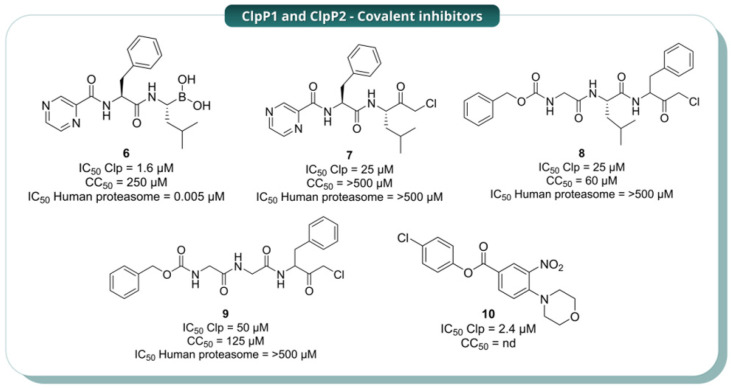
Representative covalent inhibitors of the mycobacterial ClpP1P2 complex. The figure highlights key enzymatic potency (IC_50_), cellular cytotoxicity (CC_50_), and, where available, comparative selectivity versus the human proteasome.

Given the essential role of ClpP1P2 in mycobacterial physiology, several efforts have focused on identifying small-molecule inhibitors of this complex. In 2015, Moreira et al. identified bortezomib (**6**, Figure 7) as a ClpP1P2 inhibitor through a high-throughput, mechanism-based, whole-cell screen. This was accomplished by utilising an *M. smegmatis* reporter strain that had been engineered to fluoresce when ClpP1P2 was inhibited. The screening resulted in the identification of **6** as a hit, with an IC_50_ of 6 µM and a MIC_50_ of 4 µM against *M. smegmatis*. Subsequent studies showed that the compound also exhibited antibacterial activity against the H37Rv strain of *Mtb* (MIC_50_ = 0.8 µM) and against *M. bovis* BCG (MIC_50_ = 0.3 µM). Molecular modelling further demonstrated that **6** engages the catalytic serine residues of both ClpP1 and ClpP2 through its boronic-acid warhead, forming a reversible covalent adduct that is essential for activity. The oxygen atom of the boronic acid occupies the oxyanion hole, while the other forms a salt bridge with the catalytic histidine (His123 in ClpP1 and His135 in ClpP2). Consequently, the covalent bond was substantiated by structure-activity analyses demonstrating that replacement of the boronic-acid warhead with an aldehyde resulted in the abolishment of both inhibition and antibacterial activity. Unfortunately, **6** also exhibited sub-nanomolar potency against the human proteasome (IC_50_ = 0.005 µM), thereby hindering its direct utilisation as an anti-TB agent [12]. To address this limitation, the same research group later (2017) replaced the boronic-acid warhead with a chloromethyl ketone (CMK), generating analogue **7** and related CMK derivatives **8** and **9** (Figure 7). These analogues were less potent than **6**, but they showed no measurable activity against the human proteasome up to 500 µM, indicating that improved selectivity could be achieved at the expense of potency [13].

In detail, **7** exhibited an IC_50_ of 25 µM against ClpP1P2 and a MIC_50_ of 25 µM against *Mtb* H37Rv, while **8** and **9** showed IC_50_ values of 25 and 50 µM, respectively, with MIC_50_ of 20 and 30 µM against the same strain. Toxicity profiling showed that **7** was non-toxic to HepG2 cells up to 500 µM, while **8** and **9** exhibited modest cytotoxicity (CC_50_ 60 and 125 µM, respectively), likely reflecting off-target interactions with other serine proteases. Mechanistically, the CMK moiety retains the capacity to form a permanent covalent adduct with the catalytic serine. The chlorine atom adjacent to the carbonyl creates an electrophilic centre that reacts with the nucleophilic serine residue, enabling a distinct mechanism leading to irreversible covalent modification but with improved selectivity over the human proteasome [13]. Thus, although less potent than the boronic-acid scaffold, the CMK series demonstrated that significant gains in selectivity could be achieved while maintaining covalent engagement of the protease. Compound **8** was later examined in detail by Vahidi et al. (2020) [14], who provided the first direct structural and biochemical evidence of covalent engagement with the mycobacterial protease. Electrospray Ionisation Mass Spectrometry (ESI-MS) was utilised to confirm that **8** modifies the ClpP1 ring exclusively, with no detectable reaction on ClpP2. Moreover, high-resolution cryo-EM revealed clear density caused by **8** at each of the seven ClpP1 active sites, supporting the formation of covalent adducts. In the presence of **8**, the modified subunits bind the enzyme in the R-state by ordering the handle region and re-establishing the oligomerisation-sensing salt bridge. These changes are subsequently transmitted to neighbouring unmodified protomers within the same ring. However, when all seven ClpP1 protomers are covalently modified, activity is lost because every active site becomes permanently blocked, even though the complex remains in the R-state. This behaviour is indicative of a cooperative allosteric mechanism consistent with a modified Monod-Wyman-Changeux model, in which covalent engagement of only a subset of subunits is sufficient to shift the entire ring toward activation. These observations indicate that **8** acts both as a covalent inhibitor of ClpP1P2 and as a chemical tool to probe the allosteric communication and conformational regulation of the protease. Confirming this interpretation, NMR spectroscopy data revealed new peak sets and intensity changes upon **8** modification that are characteristic of intraring allosteric communication, further supporting the notion that covalent modification induces a structural enzyme reorganisation [14]. Taken together, these data show that, although the CMK series did not surpass bortezomib as an antibacterial lead, it improved selectivity while retaining activity. It also provided important mechanistic insights into ClpP1P2 allostery, highlighting selective ClpP1 engagement as a potential strategy to control the conformational state of the complex [14].

Beyond these efforts, ClpP1P2 was also targeted by structurally diverse scaffolds developed by Xiao et al. (2025) [15], who identified **10** as a promising inhibitor in mycobacteria (Figure 7). The compound showed an initial IC_50_ of 50 µM in *M. bovis* and increased activity in a ClpP1-depleted BCG strain, where the MIC decreased from about 52.1 µM to 7.9 µM, supporting on-target susceptibility in whole cells. Biochemical validation with the purified ClpP1P2 complex showed low-micromolar potency (IC_50_ = 2.4 µM), which further improved after pre-incubation (to ~0.13 µM), consistent with near-irreversible inhibition. These data indicate that **10** is a cell-penetrant, selective covalent inhibitor that targets ClpP1, likely through acylation of the catalytic Ser98, whereas ClpP2 is not detectably modified [15]. Although this series is still at an early stage, it is notable because it supports the idea that selective covalent targeting of ClpP1 may be sufficient to impair the activity of the heterooligomeric complex [15].

Overall, the growing literature establishes ClpP1P2 as a mechanistically unusual but attractive target for covalent binding. Unlike classical proteases, its inhibition is tightly coupled to conformational control of the entire assembly, allowing covalent ligands to modulate both catalytic activity and allosteric state. Balancing potency and selectivity remains a central challenge. Importantly, selective engagement of ClpP1, as shown by **8** and **10**, indicates that full occupation of both rings may not be required for functional inhibition, pointing toward new strategies for the design of more selective ligands.

**Figure 8:**
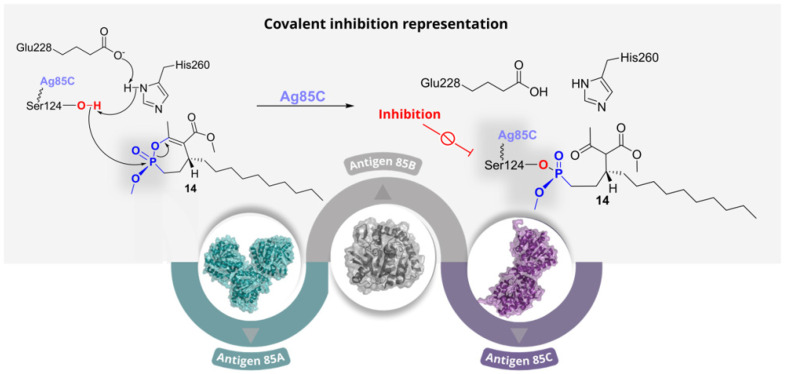
Ag85 isoforms (Ag85A in green, Ag85B in grey, and Ag85C in purple). The upper panel shows the catalytic mechanism with the formation of a stable covalent adduct with an inhibitor.

The Antigen 85 complex (Ag85A, Ag85B, and Ag85C) of *Mtb* plays a pivotal role in cell-wall biogenesis by catalysing the transfer of mycolic acids to trehalose, forming trehalose monomycolate (TMM) and trehalose dimycolate (TDM). Together, these lipids constitute a major fraction of the mycobacterial envelope and are critical for viability and intrinsic drug resistance. Because the three Ag85 isoforms share 65–75% sequence identity and retain a highly conserved catalytic machinery, they are best viewed as functionally overlapping determinants of mycolate assembly rather than fully distinct targets. Their activity depends on the conserved Ser124-Glu228-His260 catalytic triad, with Ser124 acting as the nucleophile (Figure 8). This conservation suggests that active-site inhibitors could potentially act across all three Ag85 isoforms. Moreover, because they are secreted and exposed in the extracellular milieu, they may offer advantages in terms of target accessibility. Consequently, the Ag85 complex represents an attractive extracellular and functionally redundant target for anti-mycobacterial intervention [16].

**Figure 9:**
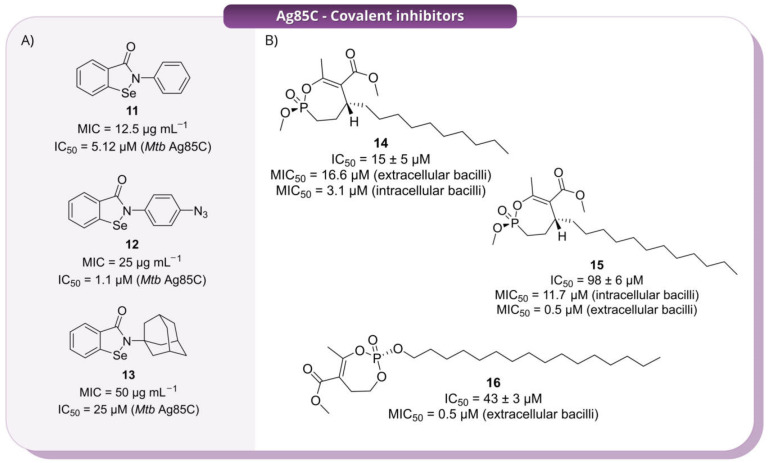
Representative covalent inhibitors reported for Ag85C. In Panel (**A**), compounds **11**, **12** and **13** are shown with their whole-cell MIC values and in vitro IC_50_ values. In Panel (**B**), compounds **14**, **15** and **16** are displayed with IC_50_ values and MIC_50_ values against extracellular or intracellular bacilli.

Ebselen (**11**, 2-phenyl-1,2-benzisoselenazol-3(2*H*)-one, Figure 9, Panel A) was initially identified as an inhibitor of the Ag85 complex of *Mtb*, suppressing trehalose dimycolate biosynthesis [17]. Subsequent studies, including a focused library of derivatives reported by Thanna et al. (2017), showed promising IC_50_ values of 12.5–100 µg mL^−1^ against Ag85, although a clear structure-activity relationship (SAR) was not established [18]. Building on these findings, Goins and co-workers (2017) investigated Ebselen (**11**) and its derivatives (Figure 9, Panel A) as covalent allosteric inhibitors of the Ag85 complex [16]. Because initial data did not provide a clear SAR, the authors selected chemically divergent analogues (**12** and **13**, Figure 9, Panel A) to systematically probe how modifications at the solvent-exposed aryl substituent influence covalent reactivity, enzyme recognition, and the conformational control of Ag85. Although IC_50_ values did not reveal a clear structure–activity relationship, kinetic analysis showed that compound **11** remained the most efficient inhibitor (*k_inact_*/*K_I_* = 0.3057 ± 0.0140 µM^−1^ min^−1^), with the azido derivative (**12**) retaining substantial activity (*k_inact_*/*K_I_* = 0.1845 ± 0.0094 µM^−1^ min^−1^) and the adamantyl analogue (**13**) displaying markedly reduced potency (*k_inact_*/*K_I_* = 0.0065 ± 0.0003 µM^−1^ min^−1^). These data proved that relatively distal structural changes can strongly influence functional inactivation. Structural analysis demonstrated that these compounds do not target the catalytic serine directly; instead, they act through covalent modification of the non-catalytic Cys209, triggering displacement of the α9-helix and mispositioning of the catalytic Glu228. Thus, inhibition results from allosteric conformational disruption rather than active-site blockage. Importantly, the similar thermodynamic favourability predicted for covalent adduct formation across the derivatives suggests that differences in inhibitory potency are driven primarily by non-covalent recognition and productive binding geometry, rather than by intrinsic selenium reactivity alone. Overall, this study establishes ebselen-based compounds as covalent allosteric inhibitors of Ag85 and highlights conformational control as a key determinant of activity [16].

In 2018, Viljoen et al. investigated a rationally designed library of 27 monocyclic cyclipostin and cyclophostin analogues (CyC) [19]. These were originally developed as selective inhibitors of mycobacterial lipases such as Rv0183 and LipY, with minimal activity against mammalian gastric and pancreatic lipases [20]. Phenotypic screening against *Mtb* H37Rv identified 8 compounds with significant anti-TB activity. Among them, **14**, **15** and **16** were selected to deepen the mechanistic analysis (Figure 9, Panel B). Compound **14** was the most active against intracellular bacilli (MIC_50_ = 3.1 μM), followed by **15** (MIC_50_ = 11.7 μM); conversely, **16** exhibited an interesting activity against extracellular bacilli, with an MIC_50_ value of 0.5 μM. All three compounds inhibited Ag85C, with **15** showing the greatest potency, followed by **14** and **16**. In cells, they impaired trehalose dimycolate formation and arabinogalactan mycolylation, with a corresponding accumulation of trehalose monomycolate, consistent with inhibition of mycolate transfer. Compound **16** also affected triacylglycerol biosynthesis, suggesting a broader effect on mycobacterial lipid metabolism. Target relevance was supported by the attenuation of these effects upon Ag85C overexpression. Importantly, these compounds were reported to be selective for mycobacterial enzymes, with no detectable inhibition of mammalian digestive lipases or the essential mycobacterial Tgs1 DGAT, and no evident toxicity toward human cells in previous studies. Taken together, these findings identify CyC derivatives as selective covalent Ag85 inhibitors while also showing that closely related compounds can display markedly different activity profiles in intracellular and extracellular settings. This is a key distinction for Ag85-directed inhibitors, as it suggests that biochemical potency alone may not be sufficient to predict activity across different physiological contexts [19].

Following the experimental validation of **15** as a potent covalent inhibitor of the Ag85 complex by Viljoen et al. (2018) [19], Adewumi and co-workers (2020) [21] examined the structural and dynamic consequences of its irreversible binding to Ag85C at the molecular level. This follow-up study was primarily computational, but it provided a useful structural rationale for the experimental activity of **15** by suggesting that covalent modification of Ser124 perturbs local loop organisation and dynamic communication around the active site. Thus, it complemented the experimental work by offering a possible explanation for how irreversible Ser124 modification propagates broader structural destabilisation in Ag85 [21].

Collectively, these studies underscore the mechanistic diversity of covalent inhibition within the Ag85 complex. On the one hand, compounds such as **11**–**13** act through allosteric covalent modification of a non-catalytic cysteine, showing that irreversible inhibition does not necessarily require direct attack on the catalytic nucleophile. On the other hand, compounds **14**–**16** follow a more classical active-site-directed strategy involving modification of Ser124. These works suggest that Ag85 inhibition is not governed solely by intrinsic warhead reactivity, but also by the ability of covalent ligands to exploit local conformational and functional vulnerabilities within the enzyme. At the same time, the variable intracellular and extracellular activity observed for the CyC derivatives highlights an important limitation for this target class and indicates that whole-cell efficacy remains strongly influenced by biological context. For this reason, future development in this area will likely depend not only on improving enzymatic potency but also on better understanding how covalent Ag85 inhibitors behave in the different microenvironments encountered by mycobacteria during infection.

### 2.2. DprE1

**Figure 10:**
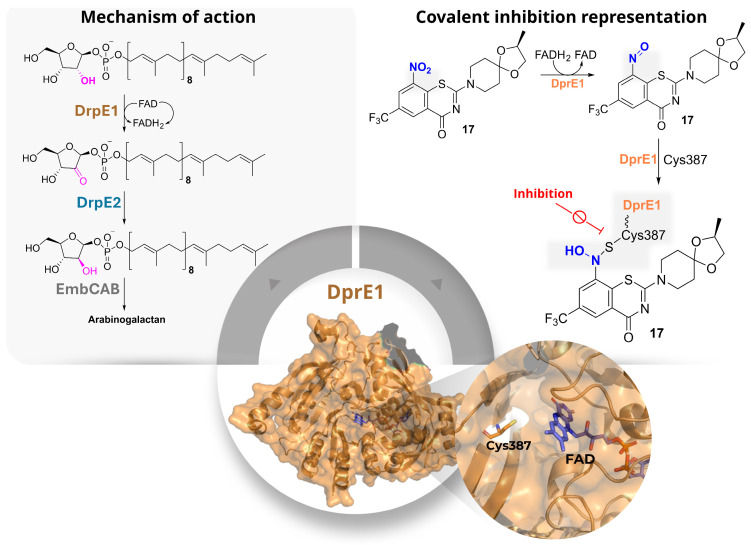
Structure, mechanism, and covalent inhibition of DprE1. The left panel illustrates the catalytic mechanism of DprE1, showing the conversion of substrates in arabinogalactan. The right panel exemplifies the mechanism of covalent inhibition of DprE1, where electrophilic inhibitors react with the catalytic cysteine (Cys387) to form a stable covalent adduct, effectively blocking enzyme activity.

DprE1 (decaprenyl-phosphoryl-β-d-ribose 2′-epimerase 1) is an FAD-dependent oxidative enzyme involved in the first step of the epimerisation process required for arabinogalactan biosynthesis, one of the three main structural components of the *Mtb* cell wall, together with peptidoglycan and mycolic acids (Figure 10). In association with DprE2, DprE1 converts decaprenyl-phospho-β-d-ribose (DPR) into the keto intermediate decaprenyl-phospho-β-2′-keto-d-ribose (DPX), which is then reduced to decaprenyl-phospho-d-arabinose (DPA), the only arabinose donor used for arabinogalactan assembly [22].

From a structural point of view, DprE1 contains a buried FAD-binding domain and a substrate-binding cavity that can accommodate both the lipophilic decaprenyl chain of the natural substrate and a variety of hydrophobic inhibitor scaffolds. The catalytic site is reached through a channel leading to Cys387 in *Mtb* (Cys394 in *M. smegmatis*), which is the key residue involved in covalent inhibition by nitroaromatic compounds. In these cases, enzymatic reduction of the nitro group generates a nitroso intermediate that subsequently reacts with the thiol of Cys387 to form a stable semimercaptal adduct (Figure 10). Because of its essential role in cell-wall biosynthesis, its accessibility, and the large number of active chemotypes identified so far, DprE1 has become one of the most extensively explored covalent targets in anti-TB drug discovery [23].

**Figure 11:**
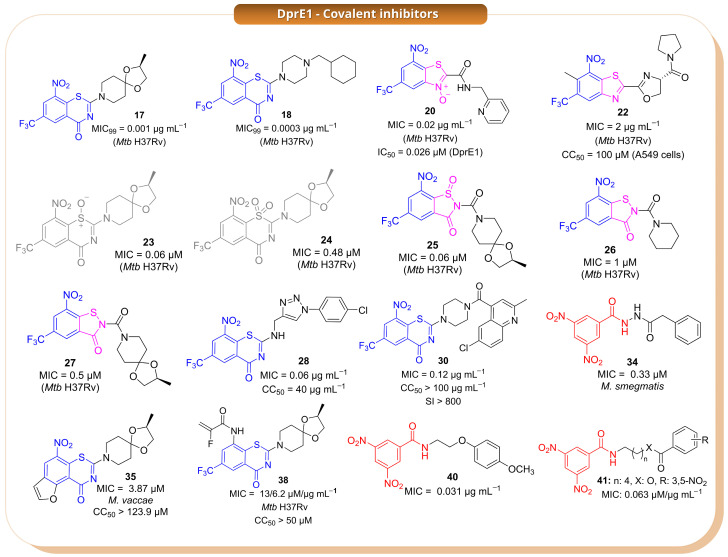
The panel compiles representative covalent DprE1 inhibitors across key chemotypes and studies. Colour coding in the chemical structures highlights the corresponding scaffolds: blue denotes the benzothiazinone (BTZ) core (e.g., **17**), pink indicates oxidised BTZ analogues (e.g., **20** and related variants), and red marks the dinitrobenzamide (DNB) scaffold (e.g., **40**). The remaining DprE1 inhibitor structures mentioned in the text are reported in Table S1.

Among the earliest and most important covalent DprE1 inhibitors are the benzothiazinones, represented by compounds **17**, **18** (Figure 11), and **19** (Table S1), which established the now classical nitro-dependent covalent mechanism involving reductive activation followed by modification of Cys387 [22,23]. Based on this precedent, Landge et al. (2015) identified the benzothiazole-*N*-oxide (BTO) series through a phenotypic screening campaign and selected compound **20** as the most promising hit (Figure 11) [24]. However, despite the favourable antibacterial profile, the BTO scaffold was affected by significant liabilities, including AMES positivity, CYP inhibition, and cellular toxicity. These findings highlighted a central issue in this area: the challenge is not only to retain strong DprE1 inhibition, but also to reduce the drawbacks often associated with nitroaromatic covalent warheads [24].

To address these limitations, the same work examined related benzothiazole derivatives lacking the *N*-oxide moiety (BTs) as well as sterically crowded benzothiazoles (cBTs). Within the BT series, compound **21** (Table S1) emerged as the most potent analogue, showing an IC_50_ of 0.3 µM against DprE1 together with a bactericidal profile [24]. By contrast, the cBT derivatives were generally less potent, but they showed a much better safety profile, including loss of mutagenicity, lower CYP inhibition, and no detectable toxicity up to 100 µM. This trade-off between potency and safety is a recurring feature in the DprE1 field, and these early studies indicate that improvements in the toxicological profile are often accompanied by reduced enzymatic activity. This issue was further investigated by Landge et al. (2016), who explored whether steric and stereoelectronic modulation of the crowded benzothiazole scaffold could improve that balance [25]. In this series, the introduction of an *ortho*-methyl group adjacent to the nitro substituent proved effective, leading to compound **22** as the most promising analogue (Figure 11). This candidate retained bactericidal activity, also under hypoxic conditions, while showing a clearly improved safety profile, being AMES-negative, non-toxic to A549 cells up to 100 µM, and less active against major CYP isoforms than related compounds. Mechanistic studies indicated that **22** still acts through the formation of a semimercaptal adduct with Cys387. Taken together, these results showed that suitable steric tuning around the nitro group can preserve the DprE1-directed covalent mechanism while reducing some of the main liabilities of the scaffold [25].

A different issue emerged from studies on the oxidation products of the benzothiazinone scaffold. Tiwari et al. (2015) initially reported compounds **23** and **24** as the sulfoxide and sulfone derivatives of **17**, respectively, and found that **23** retained strong antimycobacterial activity, whereas **24** was much less active (Figure 11) [26]. However, this structural assignment was later revised by Eckhardt et al. (2020), who demonstrated that oxidation under the reported conditions actually produced ring-contracted benzisothiazolinone-1-oxide derivatives such as **25** and **26** (Figure 11) [27]. This correction was significant not only from a structural perspective but also because it showed that substantial rearrangement of the BTZ core does not necessarily abolish antimycobacterial activity. On this basis, Richter et al. (2022) investigated related *N*-acyl benzisothiazolinones, including **25**–**27** (Figure 11), and identified them as promising analogues showing activity against *Mtb* and other mycobacterial species, together with improved physicochemical properties such as better aqueous solubility [28]. However, for this class, the proposed covalent mechanism is supported primarily by docking studies rather than direct structural evidence and should therefore be interpreted with caution.

Another important direction in the field has been the attempt to reduce the dependence of DprE1 inhibition on a single covalent interaction with Cys387, which is also the main source of mutation-driven resistance. In this context, Sahoo et al. (2022) designed BTZ–triazole hybrids intended to combine the known covalent warhead with additional non-covalent interactions in the binding pocket [29]. Among these compounds, **28** (Figure 11) emerged as the lead candidate, showing high potency against *Mtb* H37Rv and maintaining activity against drug-resistant clinical isolates. It also displayed a favourable selectivity index and bactericidal behaviour in time-kill assays. According to docking studies, the nitroso metabolite of **28** still reacts with Cys387 but is additionally stabilised by hydrogen-bond interactions with nearby residues, which may help explain its improved activity. Although this strategy does not remove the requirement for the catalytic cysteine, it suggests that reinforcing non-covalent recognition can improve the inhibitor profile. A related scaffold-merging strategy was described in another study by the same group (2022), where quinoline-4-carbonyl-piperazinyl-benzothiazinone hybrids were developed [30]. Several compounds in this series, particularly **29**–**32** (Figure 11 and Table S1), showed submicromolar MIC values; among them, **30** (Figure 11) was identified as the lead because of its potency, selectivity, and rapid bactericidal effect. Docking studies supported the same general covalent mechanism observed for classical BTZs. Overall, these hybridisation approaches suggest that the BTZ warhead can still be productively used when embedded into more elaborate scaffolds, provided that the resulting molecules retain both productive binding and suitable whole-cell activity [30].

In a separate study, Hu et al. (2022) used a combined computational approach based on bioactivity fingerprinting and structure-based virtual screening to identify new DprE1 inhibitors from a large commercial library [31]. This led to the discovery of two main hits, the sulfonamide **33** (Table S1) and the nitro-dihydrazide **34** (Figure 11), both active at submicromolar concentrations against *M. smegmatis*. While **33** was proposed to act through a non-covalent mechanism, **34** was suggested to behave as a covalent DprE1 inhibitor based on its nitro-containing scaffold and indirect evidence of target engagement, including thermal stabilisation of the enzyme. This study shows that both covalent and non-covalent chemotypes can emerge from the same discovery workflow, although in the case of **34**, the mechanistic assignment is less secure than for better characterised nitroaromatic inhibitors [31].

Scaffold diversification of the BTZ series was also explored by Keiff et al. (2023), who expanded the benzene ring of the BTZ core to generate benzofuran-fused and naphthalene-fused thiazinone analogues [32]. Compounds **35** (Figure 11) and **36** (Table S1) were the most promising representatives of these new series and retained antimycobacterial activity without detectable cytotoxicity in HeLa cells. Docking suggested preservation of the canonical covalent binding mode, with the nitro group correctly oriented toward the catalytic cysteine and maintenance of the main stabilising interactions [33]. However, redox calculations indicated that these fused analogues were more difficult to reduce than the parent BTZ scaffold, impairing formation of the reactive nitroso intermediate and likely accounting for their reduced biological potency compared to the parent compounds. These findings highlight that, for DprE1 inhibitors, maintaining geometric complementarity with the binding pocket is insufficient if the redox properties required for warhead activation are not preserved [32].

A conceptually different strategy was reported in a subsequent study by the same group, in which the essential nitro group of classical BTZs was replaced with alternative electrophilic warheads [34]. The aim was to retain covalent reactivity toward Cys387 while reducing issues linked to nitroaromatic activation. This approach led to a nitro-free BTZ series comprising different electrophilic families, among which compounds **37**–**39** (Figure 11 and Table S1) showed the most interesting profiles. Compound **39** (Table S1) displayed the best activity against *Mtb*, whereas **38** (Figure 11) was the most potent inhibitor at the enzymatic level. Additional reactivity and cytotoxicity studies indicated that some of these analogues combine acceptable stability and low thiol reactivity with a reasonable preliminary safety profile. Moreover, mass spectrometry supported covalent modification of Cys387 for **37**. Although these compounds do not yet appear to outperform the best nitro-containing DprE1 inhibitors, they provide useful proof of concept that nitro-free electrophilic warheads can still support DprE1-directed covalent inhibition [34].

Finally, building on the work of Christophe and collaborators [35], who originally reported scaffold **40** (Figure 11), Delgado et al. (2024) investigated simplified DprE1 covalent pharmacophores based on 3,5-dinitrobenzamides [36]. In this series, compounds **41**–**43** (Figure 11 and Table S1) showed submicromolar antibacterial activity and a bactericidal profile, with potency close to that of the reference compound. These simplified analogues do not yet clearly surpass the best BTZ-derived inhibitors, but they show that the essential logic of DprE1-directed covalent inhibition can be retained even in structurally reduced scaffolds [36].

Overall, the DprE1 field is one of the most advanced examples of covalent inhibitor development in *Mtb*. Several chemotypes have shown strong antibacterial potency and, in some cases, encouraging preliminary safety profiles. At the same time, significant limitations remain recurrent across the literature, including liabilities associated with nitroaromatic warheads, reliance on Cys387 for covalent engagement, and the difficulty of improving developability without compromising activity. Accordingly, the most promising strategies are not those aimed solely at increasing reactivity, but rather those in which covalent efficiency is balanced with safer warhead design, favourable redox behaviour, and enhanced non-covalent recognition within the DprE1 binding pocket.

### 2.3. Ldt_Mt1_ and Ldt_Mt2_

**Figure 12:**
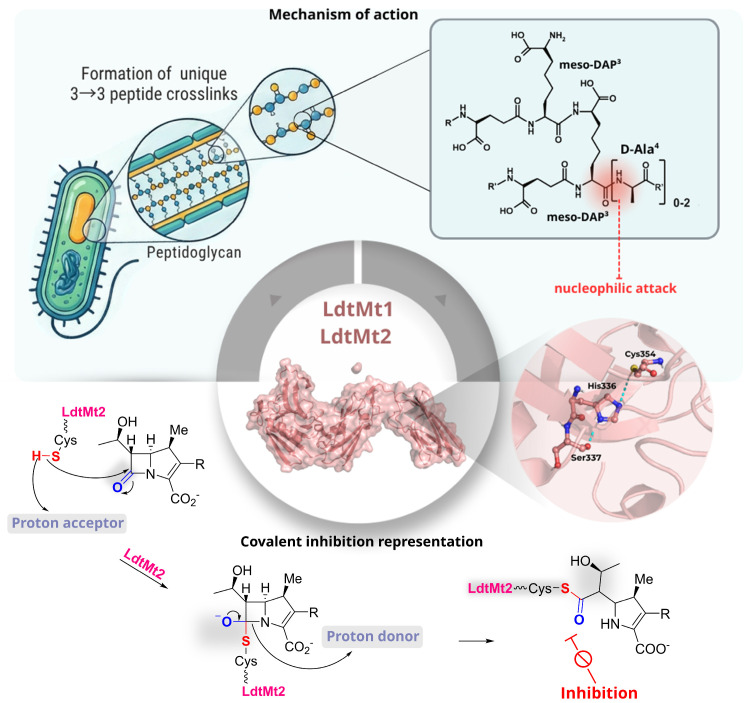
Mechanism of action and covalent inhibition of l,d-transpeptidases Ldt_Mt1_ and Ldt_Mt2_. The upper panel illustrates the catalytic function of Ldt_Mt1_ and Ldt_Mt2_ in forming unique 3→3 peptide crosslinks within the mycobacterial peptidoglycan. The inset highlights the first catalytic step, namely the nucleophilic attack by the donor meso-DAP^3^–d-Ala^4^ amide bond. The lower panel schematises the covalent inhibition.

Ldt_Mt1_ and Ldt_Mt2_ are homologous l,d-transpeptidases in *Mtb* that catalyse the formation of 3→3 peptidoglycan cross-links, a structural motif reported to predominate in stationary phase cultures and contribute to stress tolerance and antibiotic persistence [37]. Both enzymes share the conventional l,d-transpeptidase catalytic fold and rely on an essential nucleophilic cysteine, Cys226 in Ldt_Mt1_ and Cys354 in Ldt_Mt2_, to initiate catalysis through nucleophilic attack on the donor meso-DAP^3^–d-Ala^4^ amide bond, generating an acyl-enzyme intermediate that is subsequently resolved by an acceptor meso-DAP residue (Figure 12) [37].

This process is facilitated by a conserved Cys-His-Ser catalytic triad and an oxyanion-stabilising pocket that promotes tetrahedral intermediate formation. Despite the presence of a shared mechanism, the two enzymes exhibit distinct expression profiles, localisations, and active-site architectures. Ldt_Mt1_ is periplasmic and constitutively expressed, in contrast to Ldt_Mt2_, which carries an N-terminal lipobox that anchors it to the inner membrane. Furthermore, Ldt_Mt2_ is strongly upregulated in the stationary phase. Substitutions in the residues surrounding the catalytic cysteine produce active-site cavities of differing shape and polarity, thus altering peptide substrate preference and the kinetic parameters of acylation and turnover (*k*_1_ and *k*_2_/*K_app_*). It has been established that both enzymes undergo irreversible, suicide-type inactivation by carbapenems through covalent acylation of the catalytic cysteine [38]. However, Ldt_Mt2_ typically displays higher *k*_2_/*K_app_* values for the same inhibitors, reflecting a more favourable binding pocket. In line with this, loss-of-function mutants have demonstrated that Ldt_Mt2_ is more critical for maintaining cell-wall integrity and intracellular survival, thus rendering it an especially compelling target for next-generation covalent β-lactam-based anti-TB agents [39,40].

In a study by Iannazzo et al. (2016) [39], a structurally diverse carbapenem library was developed to explore covalent inhibition of the l,d-transpeptidase Ldt_Mt1_. Rather than focusing on individual compounds, the design strategy systematically varied the C2 side chain and C8 position to evaluate how steric and electronic factors influence enzyme inactivation and β-lactamase susceptibility. Among the compounds evaluated, selected analogues showed substantially improved inhibition compared with meropenem. In particular, a phenyl-triazole derivative (**44**, Table S1) and a 2-phenethyl-thio analogue (**45**, Table S1) exhibited 20–30-fold increases in *k*_2_/*K_app_*, indicating more efficient acyl-enzyme formation. At the same time, both compounds showed reduced turnover by the β-lactamase BlaC, suggesting enhanced resistance to enzymatic degradation. These results highlight a key limitation of earlier carbapenems, namely their susceptibility to β-lactamase hydrolysis, and demonstrate that targeted modifications at C2 and C8 can simultaneously improve covalent inhibition of Ldt_Mt1_ and reduce off-target enzymatic inactivation. Overall, this work illustrates how modular carbapenem design can be used to balance reactivity and stability, providing a rational framework for developing Ldt-targeting β-lactams with improved pharmacological profiles [39].

**Figure 13:**
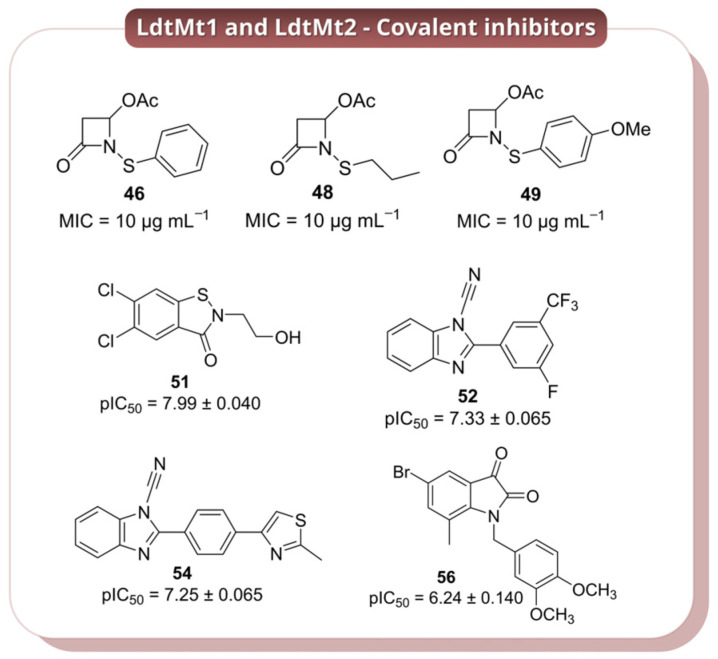
Representative covalent inhibitors of Ldt_Mt1_ and Ldt_Mt2_: the oxazolidinone-β-lactam derivatives **54**, **56** and **57** and nitrile/cyanamide compounds **46**, **47**, **49** and **51**. Additional structures are provided in Table S1.

Covalent inhibition of Ldt_Mt2_ was investigated by Martelli et al. (2021) [41], who developed a focused library of monocyclic *N*-thio-β-lactams, building on prior evidence of activity in *N*-thiolated monobactams and on the faropenem-like mechanism of enzyme trapping. Structural variations in the *N*-thio substituent were used to probe how steric and electronic factors influence reactivity toward the catalytic cysteine (Cys354). A subset of compounds (**46** > **47** > **48** > **49** > **50**, Figure 13 and Table S1) showed rapid covalent modification of the enzyme, with the most active analogues displaying reactivity comparable to meropenem. In contrast, bulkier derivatives were significantly less effective, highlighting steric constraints within the active site. The most promising candidates (**46**, **48**, and **49**, Figure 13) exhibited antibacterial activity against *Mtb*, with MIC values of ~10 μg mL^−1^ against H37Rv and retained activity against MDR strains (MIC = 20–40 μg mL^−1^), indicating that this scaffold can translate enzymatic reactivity into cellular efficacy. Mechanistically, these compounds act through an unusual disulfide exchange process, transferring small S–R′ fragments to Cys354 rather than forming classical β-lactam acyl-enzyme intermediates. This results in a stable, hydrolysis-resistant dead-end complex, consistent with irreversible inhibition. While this study highlights *N*-thio-β-lactams as an alternative covalent strategy to target Ldt enzymes, the relatively high MIC values suggest that further optimisation is required to improve potency and drug-like properties [41].

Another series of covalent inhibitors of Ldt_Mt2_ were identified by Munnik et al. (2023) [42] through a targeted HTS campaign using a library of approximately 10,000 electrophilic small molecules (MW < 450 Da) enriched in thiol-reactive motifs. Within this set, the ebsulfur analogue **51** (Figure 13) emerged as the most potent Ldt_Mt2_ inhibitor (pIC_50_ = 7.99), while the cyanamide derivatives displayed slightly lower potency but high covalent efficiency (e.g., compound **52**, *k_inact_*/*K_I_* of 265 M^−1^ s^−1^; compounds **53**–**55**, *k_inact_*/*K_I_* in the range 609–1164 M^−1^ s^−1^; Figure 13 and Table S1). Notably, several compounds showed limited activity in axenic cultures but significant intracellular efficacy in infected macrophages, with nitrile **54** (Figure 13) displaying the highest potency (MIC_50_ = 0.98 μM), followed by **51**, **52**, and **56** (Figure 13). This discrepancy highlights the importance of host-relevant conditions for evaluating Ldt inhibitors and suggests that factors such as cellular uptake, metabolic state, or target engagement within macrophages may strongly influence compound activity. Mechanistic studies confirmed covalent modification of the catalytic Cys354, with structural data (co-crystal structures with compounds **51**, **55**, **57**, **58**; Figure 13 and Table S1) supporting a consistent binding mode across different chemotypes, including proper positioning within the oxyanion hole and conformational rearrangements associated with transition-state stabilisation. This work demonstrates that electrophile-focused HTS can efficiently identify diverse covalent scaffolds targeting Ldt_Mt2_. However, the relatively high hit rate and broad reactivity of some electrophilic motifs raise potential concerns regarding selectivity and off-target effects, as reflected by the variability in cytotoxicity. Moreover, the divergence between intracellular and axenic activity underscores the need for more predictive screening models and assays to prioritise truly translatable Ldt inhibitors [42].

### 2.4. Isocitrate Lyase 1 and 2

**Figure 14:**
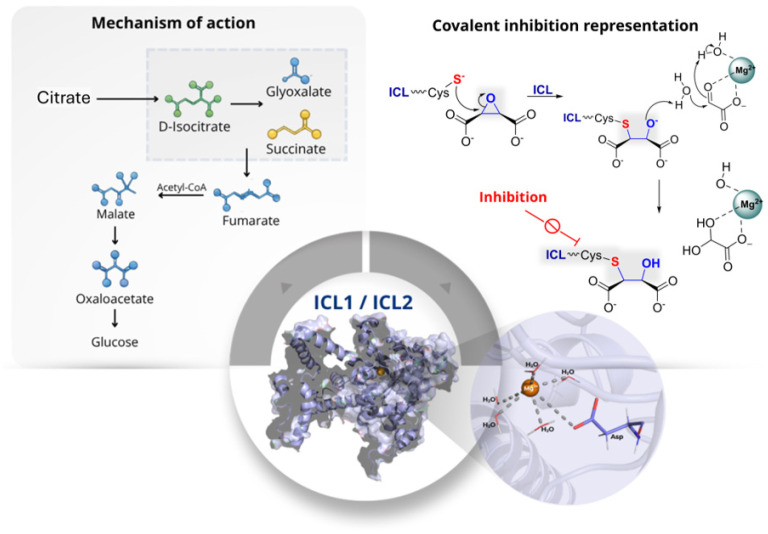
Catalytic role of ICL1/ICL2. On the top left, the schematic catalytic pathway: green metabolites denote the upstream TCA intermediate d-isocitrate, while the boxed step highlights the ICL-catalysed cleavage that yields glyoxylate (blue) and succinate (yellow); downstream is shown in blue (fumarate → malate → oxaloacetate → glucose). In the right panel, the catalytic cysteine (Cys) of the enzyme attacks the electrophilic carbon of the epoxide or other warhead of the inhibitor, forming a stable covalent adduct that blocks enzyme activity. Magnesium ions (Mg^2+^) coordinate substrate carboxylates, stabilising the transition state and facilitating nucleophilic attack.

Isocitrate lyase (ICL) is a Mg^2+^-dependent enzyme that catalyses the cleavage of isocitrate into glyoxylate and succinate, and of 2-methylisocitrate into pyruvate and succinate (Figure 14). Through these reactions, ICL initiates the glyoxylate shunt and the methylcitrate pathway and therefore plays a central role in the metabolic flexibility required for the survival and persistence of *Mtb* under host-associated conditions. In this pathogen, ICL is present as two isoenzymes, ICL1 and ICL2, which catalyse the same overall transformations but differ in size, structure, and functional contribution. The two isoforms share only 27% sequence identity, indicating a marked structural divergence [43].

Functionally, ICL1 appears to be the dominant isoform responsible for isocitrate cleavage under standard conditions, whereas ICL2 likely acts as a compensatory enzyme or contributes under specific metabolic states associated with fatty-acid utilisation. From a mechanistic point of view, ICL1 has a deep active-site cleft in which the Mg^2+^ ion is coordinated by Asp108, Asp153, and Glu182 together with substrate oxygen atoms. This arrangement organises the active site for catalysis, while Cys191 acts as a key catalytic acid/base residue during the retro-aldol cleavage that generates glyoxylate and succinate. A mobile loop governs an open-closed conformational cycle that positions the Cys191-containing segment for turnover and stabilises reactive intermediates. This catalytic organisation makes the active-site cysteine particularly susceptible to covalent inhibition. Since ICL is absent in mammals and is required for metabolic adaptation during infection, it represents an attractive target for the development of selective anti-mycobacterial agents [44].

One of the earliest mechanism-based covalent approaches against this target was reported by Pham et al. (2017), who designed the vinyl-substituted isocitrate analogue **59** (Table S1) as a putative substrate that could be enzymatically converted into an electrophilic species during catalysis [44]. More specifically, cleavage of **59** was expected to generate 2-vinylglyoxylate, a Michael acceptor capable of reacting covalently with Cys191. This design was supported by earlier affinity-labelling studies using 3-bromopyruvate. Compound **59** showed efficient time-dependent inactivation of both ICL1 and ICL2, with *k_inact_* values of 0.080 min^−1^ and 0.019 min^−1^, respectively, together with low partition ratios (0.2–0.4), indicating efficient mechanism-based inactivation. Importantly, **59** showed no cytotoxicity in human dermal fibroblasts up to 400 µM and did not react detectably with dithiothreitol (DTT), suggesting low non-specific thiol reactivity in solution. Covalent modification was confirmed by mass spectrometry and X-ray crystallography, which demonstrated formation of an *S*-homopyruvyl thioether adduct at Cys191. Overall, this study provided convincing proof of concept that catalytic activation can be exploited to generate site-directed covalent inhibition of ICL [44].

A related mechanistic framework was explored by Ray et al. (2018) [45], who re-examined the classical inhibitor 3-nitropropionate **60** (Table S1), a succinate analogue previously known as a time-dependent inactivator of ICL in different organisms. In aqueous solution, **60** coexists with its fully deprotonated conjugate base **61** (Table S1), both species acting as slow-onset covalent inhibitors with markedly different efficiencies. In particular, **61** was far more effective than **60**, with a second-order inactivation constant of 26,000 M^−1^ s^−1^ compared with 270 M^−1^ s^−1^ for the neutral form. Minimal recovery of activity after extensive dilution and the lack of reactivation by DTT supported the irreversible nature of the process. Structural and mass-spectrometric analyses confirmed formation of a stable thiohydroximate adduct at the catalytic cysteine. These findings were important because they established that nitroalkanes can function as masked electrophiles in ICL inhibition, with the nitronate form acting as the more reactive species. At the same time, the known toxicity concerns associated with nitro-containing compounds suggest that, although mechanistically informative, this scaffold may not be ideal without further optimisation [45,46].

Building on these mechanistic insights, Mellott et al. (2021) explored a substrate-based strategy designed to release the equivalent of **60** only after enzymatic turnover [47]. For this purpose, the authors synthesised (2*R*,3*S*)-2-hydroxy-3-(nitromethyl)succinate **62** (Table S1), which was intended to undergo ICL1-catalysed retro-aldol cleavage to generate glyoxylate together with a reactive nitronate species capable of covalently modifying Cys191. Compound **62** proved to be one of the most efficient mechanism-based inactivators of ICL1, with a *k_inact_*/*K_I_* value of (1.3 ± 0.1) × 10^3^ M^−1^ s^−1^ and a low partition ratio of 0.25 ± 0.03. Protection experiments with active-site ligands supported a competitive inactivation process, while mass spectrometry and crystallography confirmed formation of a thiohydroxamate adduct at Cys191. Compared with **59**, compound **62** showed substantially improved inactivation efficiency, although it remained less potent than free **60**. This result is interesting because it shows that mechanism-based substrate analogues can improve selectivity and control of reactivity, even if they do not always surpass the parent electrophile in absolute potency [47].

**Figure 15:**
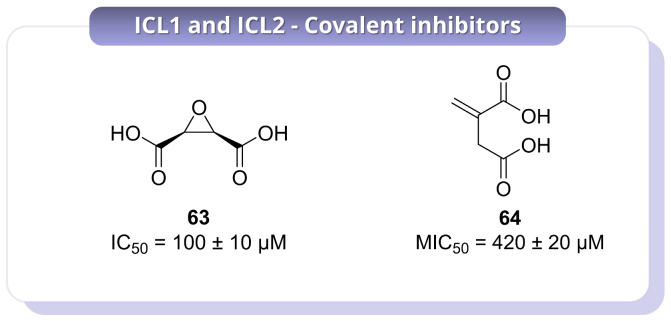
Representative covalent inhibitors of ICL1 and ICL2 reported in key studies. The structures **63** and **64** are annotated with MIC_50_ values.

Further development of substrate-mimetic covalent inhibitors was reported by Pham et al. (2021), who used a focused set of mono- and dicarboxylic acids to define the structural features required for recognition by the ICL active site [48]. This led to the identification of compound **63** as the most promising inhibitor of the series (Figure 15). Compound **63** showed potent time-dependent inactivation of ICL1, with a *K_I_* of 250 ± 10 nM and a *k_inact_* of 1.0 ± 0.1 × 10^−2^ s^−1^, giving a catalytic efficiency of inactivation of 4.0 ± 0.64 × 10^4^ M^−1^ s^−1^. By contrast, inhibition of ICL2 was markedly weaker, indicating a degree of isoform selectivity that may be useful from a design perspective. Importantly, **63** also showed antimycobacterial activity under carbon-restricted conditions, where ICL is essential, with an MIC_50_ of 100 ± 10 µM against *Mtb*, while no inhibition was observed in glucose-containing medium. This conditional activity strongly supports an on-target mechanism and emphasises the importance of metabolic context in evaluating ICL inhibitors. In addition, **63** showed no inhibition of central metabolic enzymes up to 1 mM, and proteomic profiling supported a favourable selectivity profile despite its covalent mode of action. Overall, while **63** represents an instructive example of how substrate-mimetic design can yield selective ICL inhibitors, its relatively high MIC values and the absence of mammalian cytotoxicity data indicate that further optimisation is needed to improve its translational potential [48]. A different, but biologically relevant, covalent scaffold was described by Kwai et al. (2021), who investigated the endogenous metabolite itaconate **64** (Figure 15), one of the earliest reported ICL inhibitors and a compound known to accumulate in activated macrophages [49].

In this study, **64** was shown to act as a covalent inhibitor of the mycobacterial ICL isoforms by targeting the catalytic cysteine. Covalent modification was supported by a +130 Da mass shift and was abolished in the Cys191Ser mutant, confirming residue-specific reactivity. Binding and inhibition were also found to be strongly Mg^2+^-dependent, with a dissociation constant of 112 ± 10.7 µM in the presence of Mg^2+^, compared with *K_d_* > 500 µM in its absence. Structural analysis indicated that **64** engages a network of polar interactions involving active-site residues and the metal ion, while SAR analysis showed that esterification of the carboxylate groups, as in compound **65** (Table S1), markedly reduced activity. In particular, **64** displayed an IC_50_ of 420 ± 20 µM, whereas **65** was much less active, with an IC_50_ of 2600 ± 700 µM. These results indicate that the free carboxylate groups are essential for productive binding and inhibition. However, the modest potency of **64** and its intrinsic electrophilicity raise reasonable concerns about off-target reactivity, suggesting that this scaffold is more valuable as a mechanistic and biological lead rather than as an advanced inhibitor in its current form [49].

Overall, these studies illustrate distinct strategies for covalent inhibition of ICL, including catalytic activation of latent electrophiles, masked nitro-based warheads, substrate-mimetic epoxides, and endogenous metabolite-derived inhibitors. Collectively, these approaches validate ICL as a tractable covalent target and show that selective engagement of Cys191 can be achieved in different ways. At the same time, these findings indicate that strong enzymatic inactivation does not necessarily translate into optimal whole-cell potency, and highlight metabolic context, isoform selectivity, and control of non-specific reactivity as key challenges for future development.

## 3. Conclusions

This review summarises the mechanism of action and the therapeutic potential of selected covalent inhibitors against *Mtb*, highlighting their development as an effective and established strategy in anti-TB drug discovery. Leveraging the ability of carefully designed electrophiles to form stable, often irreversible, bonds with key catalytic residues enables covalent inhibitors to achieve potent and sustained target engagement that is less dependent on systemic drug levels. Various enzymatic classes have now been convincingly validated within this framework. Serine hydrolases, including the ClpP1P2 protease and the Ag85 complex, have been successfully targeted with highly selective chemotypes. These range from chloromethyl ketone analogues that discriminate against the human proteasome to cyclipostin/cyclophostin derivatives, which exhibit submicromolar potency in vitro. DprE1, a crucial enzyme in cell wall biosynthesis, has yielded advanced covalent inhibitors, which demonstrated safety and the ability to overcome resistance. Ldt_Mt2_ is effectively inactivated by *N*-thio-β-lactams, which remain active against multidrug-resistant strains and are not hydrolysed by the β-lactamase BlaC. Finally, ICL1, a key enzyme in mycobacterial persistence, was validated using mechanism-based covalent inactivators offering both high efficiency and selectivity. Taken together, these advances demonstrate that careful optimisation of molecular scaffolds and electrophilic warheads can mitigate concerns regarding non-specific reactivity and toxicity.

## 4. Future Perspectives

Despite the significant progress achieved in the identification of covalent inhibitors targeting *Mtb* enzymes, many challenges remain to be addressed before these compounds can be translated into clinically useful anti-TB agents. Future advances in this field will require a careful balance between target binding, improved resistance profiles, high selectivity, and suitable pharmacokinetic/pharmacodynamic properties.

One of the major limitations is the potential emergence of resistance due to point mutations affecting key nucleophilic residues involved in covalent bond formation, such as Ser124 in Ag85C and Cys387 in DprE1. These mutations may markedly reduce inhibitor efficacy, highlighting the need for future design strategies that do not rely exclusively on a single covalent interaction. In this regard, reinforcing non-covalent interactions within conserved regions of the binding site may represent an effective strategy to preserve activity even in the presence of mutational changes. Approaches such as scaffold hybridisation already suggest the value of combining covalent reactivity with additional stabilising recognition elements. Another important perspective concerns the development of novel warheads with optimised reactivity and improved safety profiles. In particular, nitro-free electrophilic motifs, including Michael acceptors such as acrylamides or vinyl phosphonamidates, may offer advantages over traditional nitro-containing warheads by reducing the formation of undesirable reactive intermediates while maintaining efficient binding. More generally, the design of reversible or conditionally activated covalent inhibitors may further contribute to limiting non-specific reactivity and off-target toxicity. Selectivity also remains a critical challenge, since mycobacterial enzymes must be effectively targeted without interfering with homologous human proteins. This objective may be achieved by exploiting structural features unique to mycobacterial targets, as well as by integrating broader profiling against human homologues and the wider proteome during lead optimisation. Encouraging examples in this direction indicate that careful warhead selection and substrate-guided design can significantly improve target specificity. At the same time, greater attention should be devoted to the pharmacokinetic/pharmacodynamic implications of covalent inhibition, including intracellular accumulation, lesion penetration, metabolic stability, residence time, and tolerability. The prolonged target occupancy associated with covalent adduct formation may represent a particularly valuable feature in the treatment of TB, as it could ensure sustained target suppression even when free drug concentrations decline. This characteristic may be especially relevant against slow-growing or persistent bacillary populations, which are major contributors to the prolonged duration of TB therapy. Overall, covalent inhibitors represent a promising and still underexplored strategy in anti-TB drug discovery. Their greatest therapeutic potential may lie in their incorporation into combination regimens aimed at improving sterilising activity, limiting the emergence of resistance, and ultimately shortening treatment duration. In particular, targeting enzymes involved in metabolic adaptation and persistence, such as ICL1/2, may offer additional opportunities to address the persistent forms of infection that are difficult to eradicate with current therapies. If these challenges can be successfully overcome, covalent inhibitors may become valuable components of shorter, safer, and more effective therapeutic options for both drug-susceptible and drug-resistant TB.

## Data Availability

No new data were created or analyzed in this study. Data sharing is not applicable to this article.

## Supplementary Materials

The following supporting information can be downloaded at: https://www.mdpi.com/article/10.3390/ph19050707/s1. Table S1: Pharmacological targets, original codes, chemical structures, main biological activities, and bibliographical references of all the compounds discussed in the main text (**1**–**65**).

## Abbreviations

The following abbreviations are used in this manuscript:

NMR	Nuclear Magnetic Resonance
ABPP	Competitive Activity-Based Protein Profiling
BT	Benzothiazoles
BTO	Benzothiazole-*N*-oxide
BTZ	Nitro-benzothiazinone
cBT	Crowded Benzothiazole
CMK	Chloromethyl Ketone
CyC	Cyclipostin and Cyclophostin analogues
CYP	Cytochrome P450
DPA	Decaprenyl-phospho-d-arabinose
DPR	Decaprenyl-phospho-β-d-ribose
DprE1	Decaprenyl-phosphoryl-β-d-ribose 2′-epimerase 1
DTT	Dithiothreitol
FP-TMR	Fluorophosphonate tetramethylrhodamine
Hip1	Hydrolase Important for Pathogenesis/Rv2224c/MT2282
HTS	High-Throughput Screening
ICL	Isocitrate lyase
*K_D_*	Equilibrium dissociation constant
*K_I_*	Dissociation constant
*k_inact_*	Kinetic time-dependent inactivation
LFQ-MS	Label-Free Quantification Mass Spectrometry
*M. bovis* BCG	Live-attenuated strain of *Mycobacterium bovis*
MIC	Minimum Inhibitory Concentration
MSP-MS	Multiplex Substrate Profiling by Mass Spectrometry
*Mtb*	*Mycobacterium tuberculosis*
MW	Molecular weight
PS-SCL	Positional Scanning Synthetic Combinatorial Libraries
SAR	Structure-Activity Relationship
SH	Serine hydrolase
TB	Tuberculosis
TDM	Trehalose dimycolate
TMM	Trehalose monomycolate
WHO	World Health Organisation
