# Supplementary material for: Covalent Inhibitors Targeting Mycobacterial Enzymes: Current Status, Challenges and Future Perspectives
Source: Pharmaceuticals (Basel). 2026 Apr 30;19(5):707. doi: 10.3390/ph19050707 (PMC13209886; doi:10.3390/ph19050707)
Supplement: Supplementary file 1 [file pharmaceuticals-19-00707-s001.zip › pharmaceuticals-4195818-supplementary.pdf]

Covalent Inhibitors Targeting Mycobacterial Enzymes: Current Status, Challenges and Future Perspectives

Mariana Luiza Silva[1], Matteo Mori[2], Stefania Villa[2], Andrea Tresoldi[2], Fiorella Meneghetti[2,*] and Marcelle de Lima Ferreira Bispo[1]

[1]Department of Chemistry, State University of Londrina, Laboratory for Synthesis of Medicinal Molecules (LaSMMed), Londrina – PR, Brasil;

[2]Department of Pharmaceutical Sciences, University of Milan, Via L. Mangiagalli 25, 20133 Milano, Italy. fiorella.meneghetti@unimi.it

Supplementary Materials

Table S1. Pharmacological targets, original codes, chemical structures, main biological activities, and bibliographical references of all the compounds discussed in the main text (**1-65**).

LipO, LipI, LipH and LipG					
**Substances**	**Original code**	**Structure**	**Biological Assays**		**Ref**
			**EC_{50} (μM)**	**CC_{50} (μM)**	
**1**	JCP276	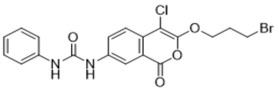	1.7 ± 0.2	112	Babin *et al.* 2022
**2**	BMB034	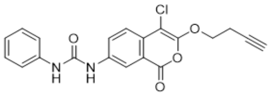	8.7 ± 1.1	N/D	
**3**	BMB038	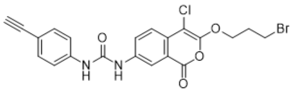	36 ± 11	N/D	

Hip1					
**Substances**	**Original code**	**Structure**	**Biological Assays**		**Ref**
			**k_{cat}/K_M ($M^{-1}s^{-1}$)**		
**4**	CSL173	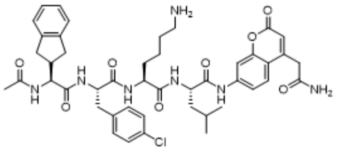	9,6 × 10^6		Lentz *et al.* 2016
			**IC_{50} (nM)**	**$k_{obs}/[I]$ ($M^{-1}s^{-1}$)**	
**5**	5	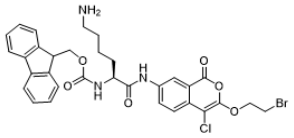	≈ 34	69,000 ± 9,600	

ClpP1P2						
**Substances**	**Original code**	**Structure**	**Biological Assays**			**Ref**
			**IC_{50} (μM)**	**IC_{50} (μM) Human Protease**	**CC_{50} (μM)**	
**6**	BZ	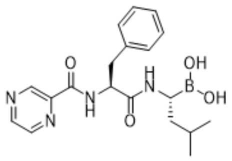	1.6	0.005	250	Moreira *et al.* 2015

**7**	Pyr-FL-CMK	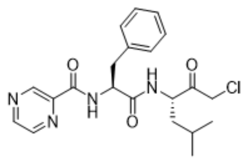	25	>500	>500	Moreira *et al*. 2017
**8**	Z-GLF-CMK	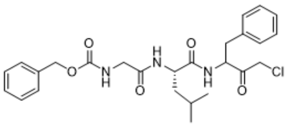	25	>500	60	Vahidi *et al*. 2020 and Moreira *et al*. 2017
**9**	Z-GGF-CMK	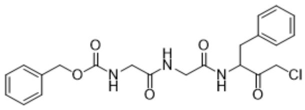	50	>500	125	Moreira *et al*. 2017
**10**	GLF-CMK	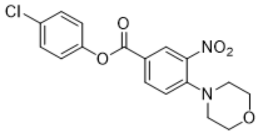	2.4	nd	nd	Xiao *et al*. 2025

Ag85C

**Substances**	**Original code**	**Structure**	**Biological Assays**		**Ref**
			**MIC *Mtb* mc²6206 (µg mL⁻¹)**	**IC₅₀ (µM)**	
**11**	Ebselen	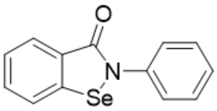	12.5	5.12	
**12**	*p*-Azido phenyl	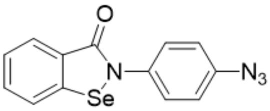	25	1.1	Goins *et al*. 2017
**13**	Adamantyl	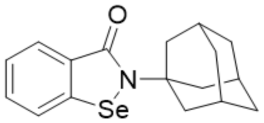	50	25	

			**Biological Assays**			
			**IC₅₀ (µM)**	**MIC₅₀ extracellular bacilli (µM)**	**MIC₅₀ intracellular bacilli (µM)**	
**14**	CyC7	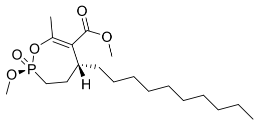	15 ± 5	16.6	3.1	Viljoen *et al*. 2018

**15**	CyC8	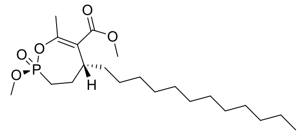	98 ± 6	11.7	nd	
**16**	CyC17	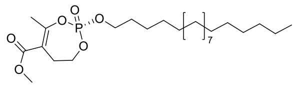	43 ± 3	0.5	nd	

**DprE1**				
**Substances**	**Original code**	**Structure**	**Biological Assays**<br>**MIC_{99} *Mtb* H37Rv (µg mL^{-1})**	**Ref**
**17**	BTZ043	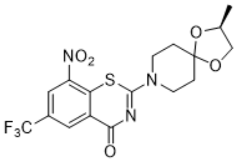	0.001	Marakov *et al*. 2014
**18**	PBTZ169	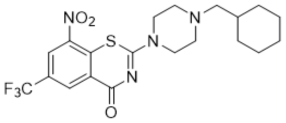	0.0003	
**19**	PBTZ129	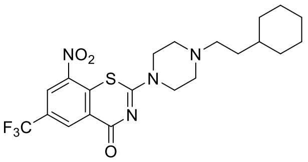	0.00019	

			**Biological Assays**		
			**MIC *Mtb* H37Rv (µg mL^{-1})**	**IC_{50} (µM)**	
**20**	9	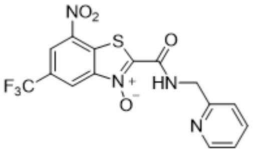	0.02	0.026	Landge *et al*. 2015

**21**	17	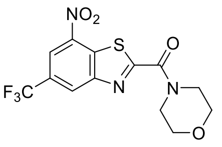	0.1	0.3	

			Biological Assays		
			**MIC *Mtb* H37Rv (μg mL^{-1})**	**CC_{50} A549 cells (μM)**	
**22**	6a	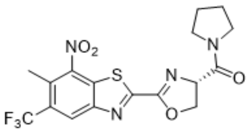	2	100	Landge *et al*. 2016

			**Biological Assays**	
			**MIC *Mtb* (μM)**	
**23**	BTZ-SO	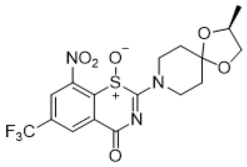	0.06	Tiwari *et al*. 2015
**24**	BTZ- SO_2	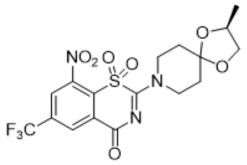	0.48	
**25**	BIT 1-oxide (4b)	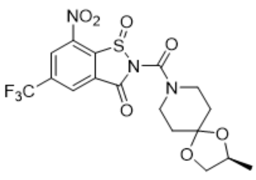	0.06	Eckhardt *et al*. 2020 and Richter *et al*. 2022
**26**	4a	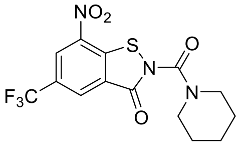	1	Richter *et al*. 2022

**27**	BIT	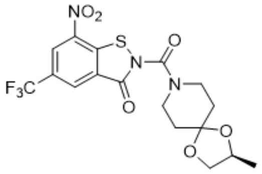	0.5		Eckhardt *et al*. 2020
			**Biological Assays**		
			**MIC *Mtb* H37Rv (μg mL^{-1})**	**CC$_{50}$ (μg mL^{-1})**	
**28**	9d	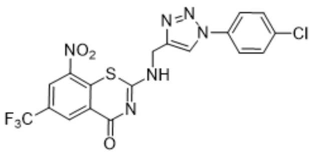	0.06	40	Sahoo *et al*. 2022
			**Biological Assays**		
			**MIC *Mtb* (μg mL^{-1})**		
**29**	8i	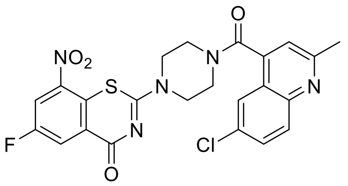	0.12		Sahoo *et al*. 2022
**30**	8aa	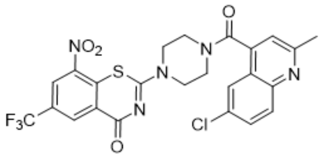	0.12		
**31**	8ab	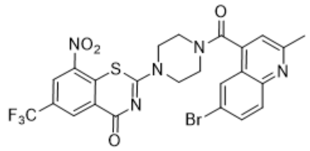	0.12		
**32**	8z	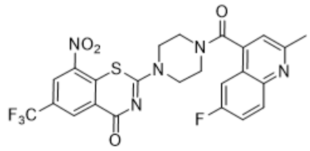	0.25		
			**Biological Assays**		
			**MIC *M. smegmatis* (μM)**		
**33**	B2	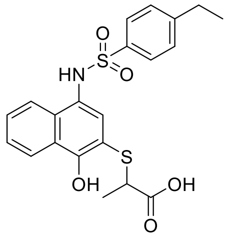	0.38		Hu *et al*. 2022

**34**	H3	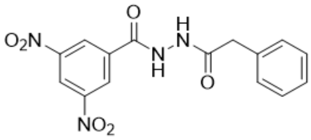	0.33			
			**Biological Assays**			
			**MIC *M. vaccae* (μM)**	**CC_{50} (μM)**		
**35**	4	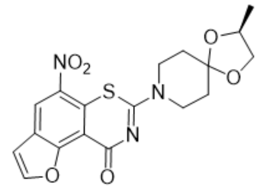	3.87	> 123.9		Keiff *et al*. 2023
**36**	5	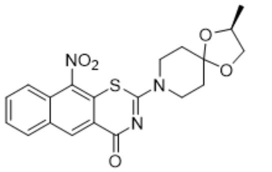	30.2	>121.1		
			**Biological Assays**			
			**MIC *M. vaccae* (μM)**	**MIC *Mtb* (μM)**	**IC_{50} (μM)**	
**37**	4e	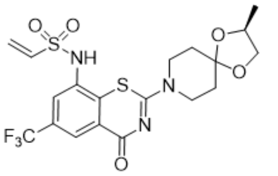	1.6	45.2	2.62 ±0.82	Torrez-Gomes *et al*. 2024
**38**	4b	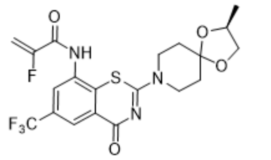	13.2	13.0	1.13 ±0.10	
**39**	4f	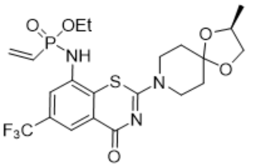	6.0	4.5	19.46 ±0.55	
			**Biological Assays**			
			**MIC H37Rv (μg mL^{-1})**			
**40**	DNB1	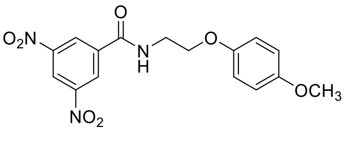	0.031			Christophe *et al*. 2009

**41**	c2	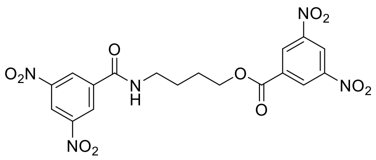	0.063	Delgado *et al*. 2024
**42**	d1	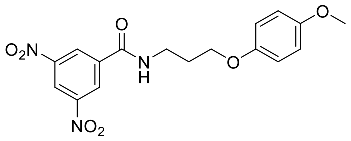	0.063	
**43**	d2	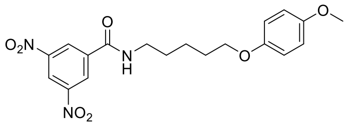	0.063	
**LdtMt1 and LdtMt2**				
**Substances**	**Original code**	**Structure**	**Biological Assays**<br>k_2/K_{app}	**Ref**
**44**	7b	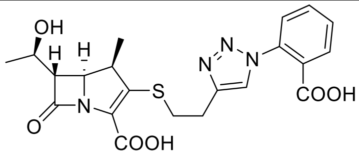	26,000 ± 1,000	Iannazzo *et al*. 2016
**45**	10c	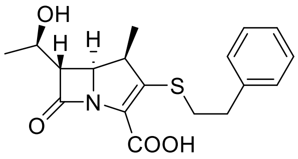	39,000 ± 1,000	
			**Biological Assays**<br>**MIC (μg mL^{-1})**	
**46**	1b	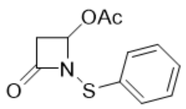	10	Martelli *et al*. 2021
**47**	1c	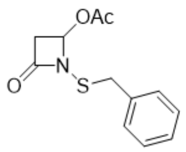	nd	
**48**	1e	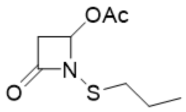	10	
**49**	1g	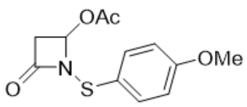	10	
**50**	1a	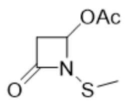	nd	

			Biological Assays			
			pIC_{50}	k_{inact}/K_I ($M^{-1}s^{-1}$)	MIC_{50} (μM)	
**51**	15	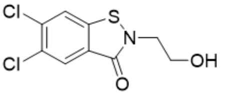	7.99 ± 0.040	nd	7.08 ± 0.16	
**52**	23	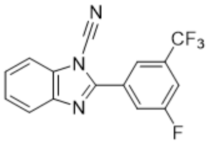	7.33 ± 0.065	265 ± 17	12.0 ± 1.5	
**53**	29	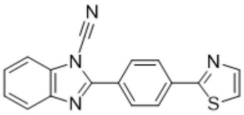	7.28 ± 0.010	1164 ± 105	nd	
**54**	30	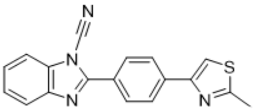	7.25 ± 0.065	236 ± 9.5	0.98 ± 1.2	de Munnik *et al*. 2023
**55**	31	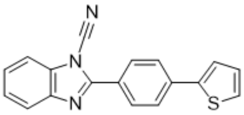	7.32 ± 0.055	609 ± 11	nd	
**56**	17	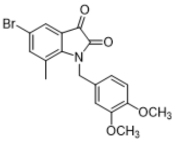	6.24 ± 0.140	nd	15.1 ± 0.70	
**57**	2	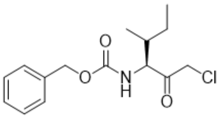	7.11 ± 0.010	68.4 ± 3.5	nd	
**58**	3	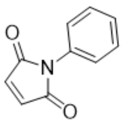	7.15 ± 0.030	3991 ± 356	nd	

ICL1 and ICL2

**Substances**	**Original code**	**Structure**	**Biological Assays**		**Ref**
			k_{inact}/K_I ICL1 ($\mu M^{-1} min^{-1}$)	k_{inact}/K_I ICL2 ($\mu M^{-1} min^{-1}$)	
**59**	2-VIC	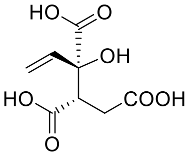	3.5 ± 0.7 x 10^{-3}	4.6 ± 0.7 x 10^{-5}	Pham *et al*. 2017
			k_{inact}/K_I ICL1	k_{inact}/K_I ICL2	

			($M^{-1}s^{-1}$)	($M^{-1}s^{-1}$)	
**60**	3-NP	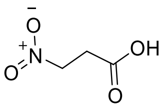	270	nd	Ray *et al*. 2018
**61**	N3P	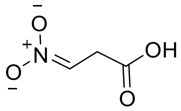	26,000	nd	
			**k_{inact}/K_I ICL1 ($\mu M^{-1} min^{-1}$)**	**k_{inact}/K_I ICL2 ($\mu M^{-1} min^{-1}$)**	
**62**	5-NIC	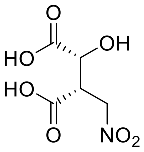	1.3 ± 0.1 x 10^{-3}	nd	Mellott *et al.* 2021
			**Biological Assays**		
			**k_{inact}/K_I ICL1 ($\mu M^{-1} min^{-1}$)**	**IC_{50} (μM)**	
**63**	Cys-EpS	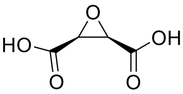	4 ± 0.64 x 10^{-3}	100 ± 10	Pham *et al.* 2021
			**Biological Assays**		
			**MIC (μM)**		
**64**	Itaconate	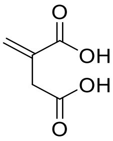	420 ± 20		Kwai *et al*. 2021
**65**	3	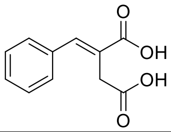	2600 ± 710		
